# Histopathologic and Prognostic Significance of Tumor Budding in Colorectal Adenocarcinoma: A Retrospective Cohort Study Conducted in Shiraz, Iran 

**DOI:** 10.30699/IJP.2023.1999329.3090

**Published:** 2021-12-15

**Authors:** Mohammad Hossein Anbardar, Nadia Rahimizadeh

**Affiliations:** 1 *Department of Pathology, School of Medicine, Shiraz University of Medical Sciences, Shiraz, Iran*; 2 *Student Research Committee, Shiraz University of Medical Sciences, Shiraz, Iran*

**Keywords:** Neoplasms, Neoplasm Metastasis, Survival Rate, Recurrence

## Abstract

**Background & Objective:**

Colorectal cancer is the second reason for cancer-associated death. The prognosis of the malignancy is defined by TNM scoring. However, tumor grading, lymphovascular invasion, perineural invasion, and tumor buddings may affect its prognosis. This study aimed to assess the prognostic and histologic impact of tumor budding in colorectal adenocarcinoma.

**Methods:**

This study is a retrospective cohort of 192 patients with colorectal adenocarcinoma. All four stages of colorectal adenocarcinoma patients were included, but the patients in stages I and II were also analyzed separately. We used pathology reports to extract the histopathologic data. The prognostic values were extracted by calling the patients.

**Results:**

Less than half of the patients were in stages I and II of the disease. According to our analysis, tumor extension and lymphovascular invasion were correlated with tumor budding count in patients in stages I and II, and lymphovascular invasion, tumor grade, tumor stage, lymph node involvement, tumor extension, tumor site, metastasis, and five-year survival were correlated with tumor budding within all stages.

**Conclusion:**

It is recommended that tumor budding count should be assessed and reported in pathology reports of adenocarcinomas due to its high correlation with poor prognosis.

## Introduction

Based on current studies, colorectal cancer is the second leading cause of cancer-related deaths globally in both genders (1). While the prognosis of malignancy is typically determined by TNM scoring, other factors can impact prognosis, such as tumor grading, lymphovascular invasion, perineural invasion, tumor border configuration, and tumor budding (2). Therapeutic management of different types of colorectal cancers also presents challenges. For example, stage II colorectal carcinomas exhibit a wide spectrum of prognoses, ranging from poor to good. Some stage II patients may require adjuvant therapy, while others may not (3, 4).

Tumor budding refers to small clusters of tumor cells, consisting of a maximum of five cells, found at the invasive surface of tumors, extending from the main malignant gland into the neighboring stroma (5, 6). Studies have shown that the presence of tumor budding is correlated with tumor metastasis (7). Additionally, detecting tumor budding in the early stages of colorectal cancers can be utilized to guide preventative surgical management (8, 9).

The aim of this study was to evaluate the prognostic and histopathologic significance of tumor budding in different stages of colorectal adenocarcinoma and its relationship with demographic, macroscopic, and microscopic findings. Furthermore, we specifically assessed these factors in stages I and II to elucidate the importance of tumor budding in the early stages. Additionally, we investigated the association between tumor budding and survival years as an indicator of disease prognosis.

## Material and Methods

**Fig. 1:**
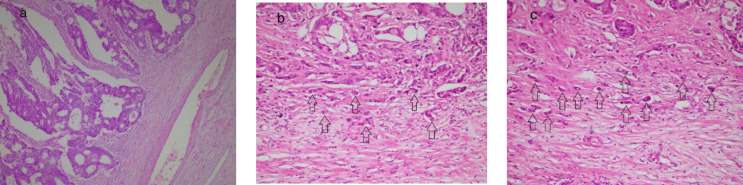
Microscopic section from the colon adenocarcinoma showing an infiltrative front of the tumor with a) no budding (Hematoxylin and Eosin, 100×); b) intermediate buds (arrows) (Hematoxylin and Eosin, 200×); c) high buds (arrows) (Hematoxylin and Eosin, 200×)

This study was a retrospective cohort conducted on 192 patients with colorectal adenocarcinoma who had undergone surgical resection from 2010 to 2017 in hospitals affiliated with Shiraz University of Medical Sciences. The inclusion criteria were all the patients with colorectal adenocarcinoma who had undergone surgical resection without previous adjuvant therapy. The patients' pathologic slides were prepared using the H&E technique; their reports were collected from the laboratory and evaluated to confirm their reports and check for tumor budding counts. The Olympus BX50 microscope with a field size of 22 mm and magnification of ×200 was used to evaluate the tumor buddings. According to the College of American Pathologists' protocol, the highest bud count in the hotspot field was assessed and then normalized by dividing the absolute count by the normalization number, which was 1.21 in this study. Then, the bud counts were rounded off and classified into three scores: low (0-4 buds), intermediate (5-9 buds), and high (10 or more buds) (10). The sample imaging slide for each group is available in Figure 1*. *Then, the demographic and histopathologic data like the patient's age, tumor size, site, extension, grade, stage, lymphovascular invasion, lymph node involvement, life status (dead/alive), survival rate, five-year survival, metastasis, and tumor recurrence were recorded. In addition, the AJCC criteria were used for tumor staging and grading (11).

Using the Chi-square method, statistical analyses were performed through SPSS software (SPSS Inc., Chicago, Ill., USA). Survival analysis was performed using the Kaplan-Meier method, and we used the Log-rank test to compare survival differences. The P-value less than 0.05 was considered significant. In addition, all three degrees of tumor budding were considered separately in assessing the correlation.

## Results

Out of 192 patients, 99 (51.6%) were categorized as having low tumor buds, 43 cases (22.4%) as intermediate, and 50 (26%) of them as high. The patients' age range was 13- 89 years, with a median of 60. 91; 47.4% were under 60, and 101 patients (52.6%) were 60 or more. The information on age and gender frequencies is shown in Table 1*.*

Three tumor grades were observed in cases, the frequencies of which are displayed in Table 2*.* Statistical analyses revealed a significant correlation between the tumor grade and budding count (*P*=0.006). Moreover, 82 cases (82.8%) of low tumor buds were grade 1. Also, 31 cases (64.6%) of grade 2 had moderate and high tumor budding. Both of the two cases (100%) of grade 3 patients presented with high tumor buds.

In this study, the sigmoid was the most common site of tumor involvement, with 65 (33.9%) out of 182 cases whose tumor involvement site was documented. Demographic information of the tumor site is shown in Table 2. There was no significant correlation between tumor budding and tumor site (*P*=0.337)

 The comparison of the tumor size showed that the median size was 5 cm; 104 (54.2%) of them were less than 5cm, and 88 (45.8%) were five cm or more. There was a significant correlation with the tumor size (*P*=019).

**Table 1:** . Correlation of the demographic and prognostic factors with the tumor budding in all stages

P-value	Number of cases	
		**Age**
**0.216**	91 (47.4%) 101 (52.6%)	**<60** **≥60**
		**Gender**
**0.740**	114 (59.4%) 78 (40.6%)	**Male** **Female**
		Five-year survival **
0.000*	43 (48/9%) 45 (51.1%)	**<5 years** **≥5 years**
		Recurrence ***
**0.288**	61 (67%) 30 (33%)	**Negative** **Positive**
		Metastasis ****
0.008*	56 (54.9%) 46 (45.1%)	**Negative** **Positive**

*Significant correlation with the tumor budding, **survival condition of 88 cases was available, ***recurrence condition of 91 cases was available, **** metastasis *condition of 102 cases was available*

Additionally, the tumor extension and tumor budding were highly associated (*P*=0.000). As a result, all 12 cases of T1 had low tumor budding. Also, 18 (85, 7%) cases of T4 had moderate and high tumor budding. In addition, 45 cases (90%) with high tumor budding were in the T3 & T4 groups. Demographic statistics of the tumor extension are shown in Table 2*.*

Analysis of the tumor stages revealed that stage III, including 67 patients (34.9%), was the most common one, followed by stages II, IV. Then I. Frequency of the stages is displayed in Table 2. There was also a significant correlation between tumor stage and tumor budding (*P*=0.001). Thus, 22 (81.5%) patients in stage I and 32 (60.4%) in stage II presented with a low tumor budding. In contrast, 31 (67.4%) cases of stage IV were associated with moderate and high tumor budding. In addition, 38 (76%) cases with high tumor budding were in stages III & IV. 

A significant correlation was also found between lymph node involvement and tumor budding (*P*=0.000). Lymph node involvement data are displayed in Table 2. Seventy five (75.8 %) cases of low budding group had no lymph node involvement. In contrast, 48 cases (66.7%) with lymph node involvement showed intermediate and high tumor budding.

 A significant correlation was also found between lymphovascular invasion and the tumor budding (*P*=0.000). The lymphovascular data are shown in Table 2. Out of all, 73 patients (73.7%) with low tumor budding showed no lymphovascular invasion, while 30 patients (60 %) with high tumor budding group demonstrated positive lymphovascular invasion. 

**Table 2:** Correlation between the histologic factors and tumor budding in all stages

P-value	Number of cases	
		**Tumor extension**
0.000*	12 (6.3%) 28 (14.6%) 131 (68.2%) 21 (10.9%)	**T1** **T2** **T3** **T4**
		**Tumor stage**
0.001*	27 (14.1%) 52 (27.1%) 67 (34.9%) 46 (24%)	**I** **II** **III** **IV**
		**Lymph node involvement**
0.000*	120 (62.5%) 72 (37.5%)	**Negative** **Positive**
		**Lymphovascular invasion**
0.000*	119 (62%) 73 (38%)	**Negative** **Positive**
		**Tumor size**
**0.019** *	104 (54.2%) 88 (45.8%)	**<5cm** **≥5**
		**Tumor grade**
0.006*	142 (74%) 48 (25%) 2 (1%)	**Grade1 (well differentiated)** **Grade2 (moderately differentiated)** **Grade3 (poorly differentiated)**
		**Tumor site**
**0.337**	12 (6.6%) 24 (13.2%) 11 (6%) 15 (8.2%) 120 (65.9%) 10 (5.2%)	**Cecum** **Ascending colon** **Transverse colon** **Descending colon** **Recto-sigmoid** **Not available**

*significant correlation with the tumor budding

Out of 192, 102 patients presented with metastasis. The frequency of the cases is shown in Table 1.

The correlation with tumor metastasis was significant (*P*=0.008). Thirty five (70%) cases with a low tumor budding showed no metastasis. In contrast, in intermediate and high tumor bud groups, the rate of metastasis was higher. Seventeen out of 26 cases with intermediate tumor budding (65.4%) were positive for metastasis. In addition, 14 out of 26 cases of high tumor budding, (53.8%) showed metastasis.

Tumor recurrence was evaluated for 91 cases. Only 30 (33%) cases were positive for recurrence, and the correlation with the tumor budding was insignificant (*P*=0.288). The frequency of the cases is shown in Table 1.

**Fig. 2:**
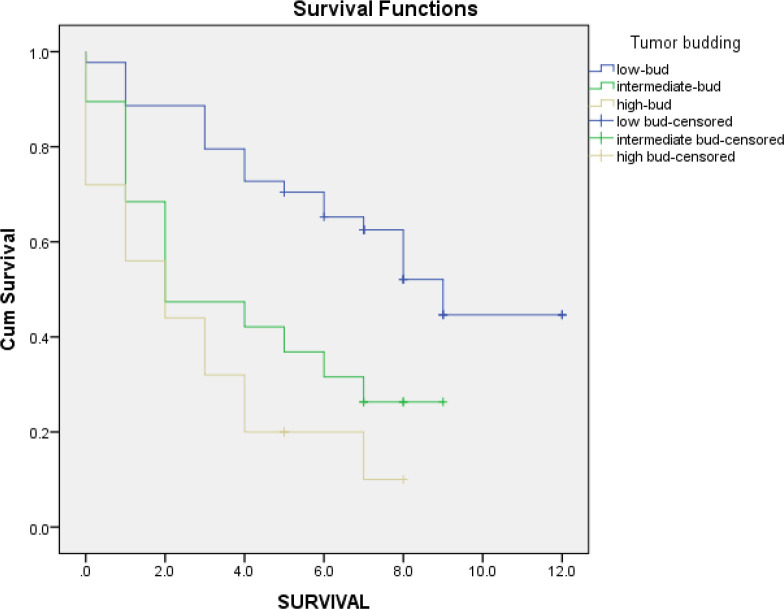
Kaplan-Meier curve: Comparing the survival rate differences in three groups of tumour budding grade in patients in all stages

Out of 88 cases in which survival rates were assessed, 43 (48.9%) had a survival rate of less than five years, and 45 (51.1%) had a survival rate of 5 or more than five years. Correlation with the tumor budding was also significant (*P*=.000). Out of 25 cases of high tumor budding, 20 (80%) had a survival rate of less than five years; in 31 (72.1%) cases with a survival rate of less than five years, an intermediate or high tumor budding was noted. In the cases with a survival rate of 5 or more than five years, 32 (71.1%) presented with low tumor budding. Besides, according to the Kaplan-Meier curves, the overall survival rate of the low tumor budding group was better than the others. Moreover, the survival rate differences in the three groups of tumor budding were also significant according to the log-rank test (*P*=000). Survival rate differences are shown in Figure 2*. * The mean survival of all the patients was around six years; also, the mean survival of the low, intermediate, and high bud groups was about eight, four, and three years, respectively. Assessment of the prognostic factors is outlined in Table 1*.*

We also analyzed prognostic data of the patients in stages I & II separately. Of all the patients, 27 (34.2%) were in stage I, and 52 (65.8%) were in stage II. Comparison of the correlation between tumor budding and stage (*P*=0.124), recurrence (*P*=0.629), and five-year survival (*P*=0.382) were not significant in the cases being in stages I & II. However, the correlation of the tumor budding with lymphovascular invasion (*P*=0.029), survival rate (*P*=0.009), and tumor extension (*P*=0.032) was significant in those two stages. As a result, 45 (72.6%) patients with no lymphovascular invasion had low tumor budding out of 62 patients in stages I and II without lymphovascular invasion. In addition, 9 (52.9%) patients who were positive for lymphovascular invasion showed intermediate or high tumor budding. In tumor extension assessment of stages I &II cases, out of 12 cases with high tumor budding, 9 (75%) were T3 and T4, and all 10 T1 patients demonstrated low tumor budding. 

**Fig. 3:**
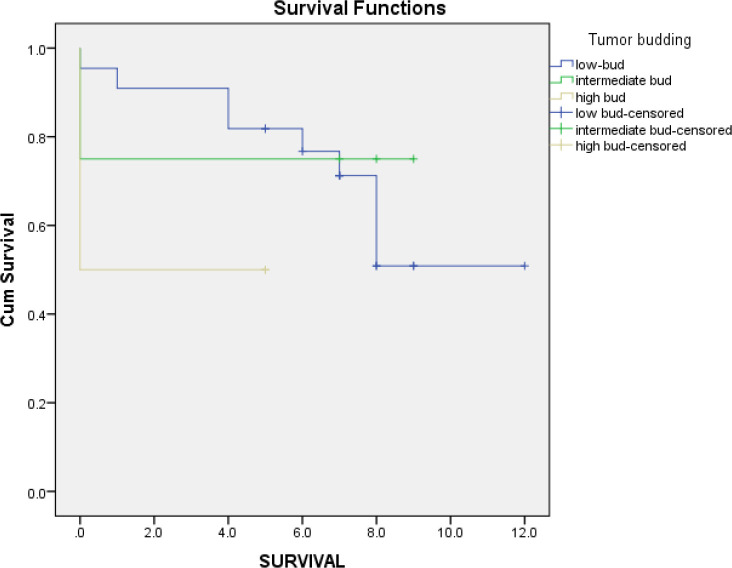
Kaplan-Meier curve: Comparing the survival rate differences in three groups of tumor budding in patients in stages I and II

Out of all 79 patients in stages I & II, survival data of only 30 patients was available. According to the log-rank test, in comparison to the overall survival rate between the three groups of tumor budding, no significant results were found (*P*=0.334). The Kaplan-Meier curve of comparing the survival rate differences is shown in Figure 3*.* The overall mean survival year for these stages was eight years. Evaluation of the factors for stages I and II are summarized in Table 3*.*

**Table 3:** Histologic and prognostic correlation with the tumor budding in stages I and II

	Number of cases	P value
Tumor stage I II	27 (34.2%) 52(65.8%)	0.124
Tumor extension T1 T2 T3 T4	10(12.7%) 16(20.3%) 49(62%) 4(5.1%)	0.032*
Recurrence Negative Positive	26(78.8%) 7(21.2%)	0.629
Lymphovascular invasion Negative Positive	62(78.5%) 17(21.5%)	0.029*
Five-year survival <5 years ≥5years	7(23.3%) 23(76.7%)	0.382
Survival rate		0.009*

* Significant correlation with the tumor budding

## Discussion

This study aimed to evaluate demographic, histopathologic, and prognostic factors of colorectal adenocarcinoma at Shiraz University of Medical Sciences, a referral center in south Iran, from 2009 to 2017. Additionally, we analyzed tumor budding in these cases and its correlation with other factors. 

In our study, T3 was the most common tumor extension, consistent with findings from other studies, such as Mehta* et al.* and Sevda* et al.* (12, 13). While some similar studies reported no significant correlation between tumor budding and tumor extension, we found a high association between these two parameters, consistent with Ueno* et al.* and Zlobec* et al.*'s studies (9, 14). Furthermore, even when we assessed tumor extension only in stages I and II, a correlation remained significant, contrary to the results of Nakamura's cohort study on 200 patients and Lai* et al.*'s research on 135 patients in stage II, which showed no significant correlation with the tumor extension (15, 16). 

In assessment of the tumor grade and budding, we observed a significant correlation between these two factors that is consistent with the results of Sevda *et al.*'s study but not those of Mehta* et al.* and Graham. Zlobec* et al.* also demonstrated a significant correlation between tumor grade and tumor budding amomg all three grades (12-14, 17). However, when categorizing patients based on tumor size, we found a high association with tumor budding, contrasting with the findings of Mehta* et al.* and Lai* et al.*'s studies (13, 15).

Lymphovascular invasion was assessed in all four stages, and we found a significant correlation between lymphovascular invasion and the tumor budding in our study, consistent with Mehta's study (13). Furthermore, in stages I and II, this correlation remained significant; same results were also concluded by Wang* et al.* and Lai* et al.*'s studies (15, 18). However, this finding contradicted results of the studies conducted by Horcic* et al.*, Nakamura* et al.*, and Jagadale* et al.*, who revealed no significant association between the mentioned factors (16, 19, 20). 

Regarding metastasis, although some studies have shown that a higher tumor budding increases the chance of recurrence and distant metastasis, Mehta's study found no relationship between these factors. Additionally, Nakamura* et al.*'s study considered tumor budding as an independent factor in predicting lung metastasis. In our study, we attempted to assess both nodal metastasis and metastasis to different organs. In contrast to Mehta* et al.*'s study, which showed no significant relationship between tumor budding and metastasis, our study revealed a significant correlation between tumor budding and the likelihood of distant metastasis (3, 13, 21-24). Additionally, the correlation was significant when analyzing the tumor budding and nodal involvement, as stated in previous studies (13, 14, 25).

Regarding recurrence, our findings were in line with Mehta* et al.*'s study, showing no significant association (13). Similarly, in stages I and II, this study's correlation was not significant, contrary to the Nakamura's and Mitrovic's studies (16, 26). This difference may be attributed to the smaller sample size in our study. Additionally, Rogers* et al.* conducted a study on patients with rectal adenocarcinoma and found a correlation between tumor recurrence and tumor budding (27). This divergent result could be due to the difference in the site of adenocarcinoma.

Regarding survival rate differences, the survival rate of the low tumor budding group was better in Mehta* et al.*'s study. Still, the five-year survival was not correlated with tumor budding (13). There is also evidence from retrospective and prospective studies indicating that presence of high tumor budding in stage II colorectal carcinomas reflects poor survival (23, 26, 28, 29). Ryan* et al.* also conducted a prospective cohort study on all stages of colorectal cancer, which revealed a worse five-year survival rate of higher budding (30). Our study compared tumor budding within all stages of colorectal carcinoma and showed that the correlation was significant with both survival rate and five-year survival. In cases with higher counts of tumor buds, survival years were lower independently from the tumor stage and vice versa. Also, assessment of five-year survival and survival rate differences for patients in stages I and II revealed that the results were not meaningful, in contrast to Nakamura* et al.* and Lai* et al.*'s studies (15, 16).

Demographic factors, such as age, sex, and tumor site, were correlated with the tumor budding neither in this study nor in other previously reported studies (15, 16).

## Conclusion

To sum up, tumor budding seems to be a significant prognostic factor in colorectal adenocarcinoma. This study found a correlation of tumor budding with nodal involvement, tumor stage, grade, extension, lymphovascular invasion, metastasis, and five-year survival. Moreover, in early stages of the colorectal cancer, this factors seems to be a prognostic factor as well for re-evaluation of the patients and categorize them in groups of high or low risk. Thus, assessment and reporting of a tumor budding count by pathologists in colorectal adenocarcinoma is highly advised. Further prospective studies with larger sample sizes are needed to assess the prognostic factors in stages I and II. It will also be challenging to assess the role of tumor budding in other types of carcinomas.

## Funding

This research received no specific grant from any funding agency in the public, commercial, or not-for-profit sectors.

## Conflict of Interest

The authors declared no conflict of interest.
